# Risk Factors for Soil-Transmitted Helminthiasis in Preschool Children Living in Farmland, North Sumatera, Indonesia

**DOI:** 10.1155/2018/6706413

**Published:** 2018-04-04

**Authors:** Sri Novianty, Yazid Dimyati, Syahril Pasaribu, Ayodhia Pitaloka Pasaribu

**Affiliations:** Department of Child Health, Faculty of Medicine, Universitas Sumatera Utara, Medan, Indonesia

## Abstract

**Background:**

Disease burden from soil-transmitted helminthiasis (STH) is mainly attributed to its chronic and insidious impact on health and quality of life. Strategy recommended by World Health Organization (WHO) to control it was previously focused on school-aged children, but now preschool-aged children are involved. This study was intended to determine STH infection risk factors in preschool children.

**Methods:**

A cross-sectional study was conducted in Suka Village, North Sumatera, Indonesia, from October to December 2016. Subjects were children aged 1 to 5 years without history of taking antihelminthic. Subjects were obtained by consecutive sampling. Demographic data and risk factors for helminthiasis were collected using questionnaire-based interview. Subjects were divided into two groups, positive and negative STH infection, based on Kato Katz method. Analysis was done using chi-square and logistic regression test. *p* value < 0.05 was considered significant.

**Results:**

We enrolled 90 subjects in this study, with the mean age being 31.7 months. STH infection prevalence was 34.4%. Statistical analysis revealed that mother/caregiver hand washing habit (*p* = 0.007), mother/caregiver nail trimming habit (*p* = 0.018), and children nail trimming habit (*p* = 0.022) were significant risk factors for STH infection.

**Conclusion:**

Mother/caregiver hand washing habit is the most influential risk factor for STH infection in preschool children.

## 1. Introduction

Intestinal parasite infections are global endemic disease and are the leading cause of morbidity worldwide [1]. Intestinal parasite infections are major health problem in developing countries, especially in children, which often cause mortality and morbidity [2]. The main cause of intestinal parasite infection is soil-borne worm called soil-transmitted helminths (STH), roundworm (*Ascaris lumbricoides*), whipworm (*Trichuris trichiura*), and hookworm (*Ancylostoma duodenale* and* Necator americanus*) [1–4].

The World Health Organization (WHO) estimates that more than two billion people with STH infection in the world experience severe morbidity, causing 9000 to 135000 deaths per year [3, 5]. Although STH infection occurs in all age groups, the WHO stated that children aged 1 to 4 years are the part of population at high risk of morbidity from STH infection [5, 6]. Diseases caused by STH infection are associated with chronic and asymptomatic morbidity in children [5–7]. Morbidity associated with STH infection includes iron deficiency anemia, malnutrition, growth and developmental disorders including short stature, and cognitive developmental disorders. Effects on the growth of children are caused by changes in appetite, digestion, absorption of nutrients, and iron deficiency. The impact of STH infection leads to poor school performance and attendance so that when they reach their adulthood their productivity tends to decrease and their pregnancy tends to be harmful, which in turn impairs the progress of children's education and the nation's economic development [6–8].

The major risk factors for STH infection are rural areas, low socioeconomic status, poor sanitation, poor availability of clean water, and poor personal hygiene [1, 8]. One of the risk factors for STH infection in preschool children is poor hygiene of mother or caregiver and child; a child's playground and a densely populated home environment may lead to the transmission and spread of the disease [2].

The prevalence of STH infection in children in Indonesia is generally very high, with the average prevalence of STH infection in Indonesia between 2002 and 2009 being 31.8% [9]. The incidence of STH infection in children from a study in Suka Village, Tiga Panah District, Karo Regency, North Sumatera Province, in 2004 was 91.3% [10]. The occurrence of STH infection was about 12.3% in children under five years old based on research results in Tegal City in 2010. There was association between the incidence of STH infection and the condition of the water supply facilities, the condition of fecal disposal facilities, the condition of the waste water disposal facilities, and the type of flooring of the house [11]. The incidence rate of STH infection in children is quite high, but there are only a few research studies related to STH infection conducted in Indonesia which assess the epidemiology and risk factors for the incidence of STH infection in preschool children as a population at risk of acquiring STH infection, and indispensable for controlling the morbidity of STH infection is a strategy recommended by WHO and the Indonesian government [5, 8, 9], especially in North Sumatera Province. This study was intended to determine risk factors for soil-transmitted helminthiasis in preschool children.

## 2. Methods

### 2.1. Study Design and Area

This was a cross-sectional study conducted in Suka Village, Tiga Panah District, Karo Regency, North Sumatera Province, in October–December 2016. Consecutive sampling was done in children aged 1–5 years, and a total sample of 90 children was obtained. The inclusion criteria were children aged 1–5 years who live in Suka Village, Tiga Panah District, Karo Regency, North Sumatera Province, and because the sample was not sufficient, other samples were taken from two other villages in Tiga Panah District, which were Suka Sipilihen Village and Suka Mbayak Village. Samples who had history of taking anthelmintic ≤1 month before the study were excluded. The number of preschool children was 108, of which 90 met the inclusion criteria, while 18 were excluded because 16 of them did not return the pots of feces, one refused to take part in the study, and the other one took antihelminthic drug in the previous month.

### 2.2. Data Collection and Laboratory Analysis

Samples which fulfilled the inclusion criteria were obtained during the Primary Health Care schedule and then interviewed to fill the questionnaire for the mother/caregiver to determine sociodemographic data of the samples and the risk factors for STH infection. The data were collected by a trained research team.

A fresh stool sample was collected from each study sample using prelabeled clean and dry formalin containing pot and then examined by Kato Katz method, and worm intensity was calculated by a certified analyst. Infected children were given appropriate therapy. Albendazole tablets (Kimia Farma, Bandung, Indonesia) were used in this study as anthelmintic treatment. Dose of Albendazole tablets for children under 2 years old was 200 mg single dose and for children aged 2 years and older was 400 mg single dose.

### 2.3. Statistical Analysis

Data were analyzed using SPSS. Nominal variables are described in frequency and proportion whereas numerical variables will be described in the mean and standard deviations or the median, minimum, and maximum values. Bivariate analysis was done using chi-square to obtain the association between the variables that were risk factors for the STH infection. Multivariate analysis was performed using logistic regression test, and *p* < 0.05 was considered significant.

### 2.4. Ethical Approval

This study has been approved by the Health Research Ethical Committee of Medical Faculty, Universitas Sumatera Utara (number: 775/TGL/KEPK FK USU-RSUP HAM/2016).

## 3. Results

The baseline characteristics of study subjects showed that the subjects of the study were dominated by girls 58.9%, with mean age of 31.7 months with the youngest subject being 12 months and the oldest being 59 months old; the mean child weight and height were 11.64 kg and 83.26 cm, respectively, with normoweight as the most frequent nutritional status 90%. Most of the fathers' occupations are in the vegetable farming category, 86.7%, as well as the mothers', 86.7%. Baseline characteristic data of the research subject is shown in Table 1.

The prevalence of STH infection was 34.4% (Table 2). A total of 31 preschool children of positive STH infection were given Albendazole therapy at the recommended dosage. The proportion of STH infection found in stool examination is found in Table 2 where proportion of* Ascaris lumbricoides* (*A. lumbricoides*) infection is 20%,* Trichuris trichiura* (*T. trichiura*) infection is 4.4%, and mixed infection is 10%. Based on the intensity of* A. lumbricoides*, we found 22.4% samples having light-intensity infection and 4.4% samples having moderate-intensity infection. Based on the intensity of* Trichuris trichiura* infection, only light intensity was obtained at 14.4% samples.

Table 3 showed the habits of children and mother/caregiver that affect the incidence of STH infection in preschool children. From the table we can see that the habit of nail trimming and hand washing of children were significantly associated with the incidence of STH infection (*p* value 0.007 and 0.003, resp.). Children who did not conduct nail trimming tend to have STH infection at 5.1 times (PR: 5.1, 95% CI: 1.57–16.83) compared to the children who did nail trimming. Children who did not conduct hand washing tend to have STH infection at 5.9 times (PR: 5.9, 95% CI: 1.62–21.89) compared to the children who did hand washing. We could also see that hand washing habit of mother/caregiver, eating uncooked food, and nail trimming habit of mother/caregiver have a statistically significant association with the incidence of STH infection in preschool children (*p* value 0.004, 0.016, and 0.018, resp.). Children from mother/caregiver who did not do hand washing will have higher probability of having STH infection at a rate of 5.3 times (PR: 5.3, 95% CI: 1.65–17.12) compared to children from mother/caregiver who did hand washing. Children from mother/caregiver who have a habit of eating uncooked food in the family have higher risk of STH infection by 4.0 times (PR: 4.0, 95% CI: 1.24–12.99) compared to the opposed group. Children from mother/caregiver who have habit of not trimming nails tend to have higher incidence of STH infection at a rate of 3.5 times (PR: 3.5, 95% CI: 1.27–9.66) compared to children from mother/caregiver who did nail trimming. The association between habits of children and mother/caregiver with STH infection in preschool children was shown in Table 3.

Table 4 shows that the multivariate logistic regression analysis test was conducted three times. This is because the test used is logistic regression with enter method, which means the regression test will be done until all the variables that have a meaningful correlation are found. Based on multiple logistic regression test after three steps, we found that hand washing habit of mother/caregiver (PR: 5.8, 95% CI: 1.63–20.75), nail trimming habit of mother/caregiver (PR: 4.1, 95% CI: 1.28–12.97), and nail trimming habit of children (PR: 4.5, 95% CI: 1.24–16.57) are the risk factors for soil-transmitted helminthiasis in preschool children.

## 4. Discussion

Soil-transmitted helminthiasis (STH) infection is a chronic infection that remains a major health problem in the world in many tropical and subtropical regions, especially in developing countries [3, 6]. In Indonesia, STH infection, which is a neglected tropical disease, is still a major health problem especially in rural areas [3], where the population of school-aged and preschool children experience the most morbidity [5–7]. The latest strategy recommended by WHO 2012 is to control STH infection morbidity in preschool age children as one of the at-risk populations [5].

This study reported the prevalence of STH infection in preschool children by 34.4% in Suka Sipilihen Village, Suka Village, and Suka Mbayak Village, Tiga Panah District, Karo Regency, North Sumatera Province. This was higher than previous studies in other parts of Indonesia by Altiara, where the prevalence in Tegal City in 2010 was 12.3% [11]. However, this study was consistent with several other studies showing the high prevalence of STH infection. A cross-sectional study in China in 2010 by Wang et al. reported that there was STH infection prevalence of 21.2% in preschool children [12]. As with previous study, Ethiopian studies in 2014 by Shumbej et al. showed STH infection prevalence of 23.3% in preschool children [6]. Zulkifli et al. in 1999 reported a higher prevalence of STH in preschool children (56%) in Kelantan, Malaysia [13].

A cross-sectional study in Sri Lanka in 2013 by Galgamuwa et al. reported a high prevalence of* A. lumbricoides* infection of 37.8% in preschool children residing in plantation areas [14]. The location of this study is consistent with the previous study of plantation areas where most mothers' and fathers' occupations were vegetable farming (86.7% and 86.7%, resp.). Previous study in Tegal City in 2010 by Altiara also reported the distribution of the type of worms that infect children under five years, with the highest incidence of infection being with* A. lumbricoides* (7.5%) and* T. trichiura* (2.5%), with no incidence of hookworm [11]. This study is consistent with previous study, where the proportion of the incidence of STH infection based on the type of worm with the highest incidence of infection is* A. lumbricoides* (20%) followed by mixed infection (10%) and* T. trichiura* (4.4%), with no hookworm incidence. However, Shumbej et al. in Ethiopia in 2014 reported that the most frequent STH infections in preschool children were caused by* A. Lumbricoides* (14.9%) followed by* T. Trichiura* (6.4%) and hookworm (3.2%) [6].

The morbidity associated with STH infection is associated with the intensity of infection [6]. The lack of focus on preschool children is based on the low prevalence and intensity of the assumed infection, coupled with the belief that low-intensity infections do not produce significant morbidity [15]. Shumbej et al. found a light-intensity STH infection in* A. lumbricoides* (98%),* T. trichiura* (95.8%), and hookworm (91.6%); moderate-intensity infections in* A. lumbricoides* (2%),* T. trichiura* (4.2%), and hookworm (8.4%); and no heavy intensity of STH infection [6]. This was in accordance with the results of this study. The most common STH infection was* A. lumbricoides* with light intensity (24.4%) but no heavy intensity. However, this study also does not accord with some previous studies. Wang et al. in China in 2010 reported finding STH infection in preschool children with light, moderate, and heavy intensity [12]. Similarly, a study in Kelantan, Malaysia, in 1999 by Zulkifli et al. reported 8.3% children suffering from heavy intensity of* A. lumbricoides* infection and 1.2% children suffering from heavy intensity of* T. trichiura* infection [13].

The prevalence of STH infection is closely linked to environmental factors and socioeconomic conditions [13]. The geospatial distribution of STH is influenced by various factors such as poor environmental sanitation, lack of personal hygiene, using contaminated water, and other factors including age, socioeconomic status, and occupation [6]. Most infections with intestinal parasites are more severe in children than adults, one of which is related to poor parenting [2]. A study in Kenya by 2016 by Worrell et al. reported high STH prevalence, 40.8%, in preschool children with one of the risk factors for STH infection being a caregiver role, with only 8.9% caregivers washing their hands before feeding children [16]. The results were in accordance with this study assessing factor individual sanitation and hygiene risks including the habits of children and mother/caregiver with the incidence of STH infection in preschool children.

In this study, the risk factors of mother/caregiver habits have significant association with the incidence of STH infection in preschool children who were not washing their hands (45%), eating uncooked food (42.2%), and untrimming nails (57.1%). This is in accordance with some previous studies. A study in Ethiopia, done by Aleka et al. in 2015, found that intestinal parasite infections were common in older children with no hand washing habit (71%), parents who lack trimming nails habit (61%), and habit of eating raw food (19.4%) [2]. Galgamuwa et al. in Sri Lanka in 2016 reported high prevalence of* A. lumbricoides* infection in children who consumed unwashed fruit by 53.1% [14].

In this study it is also found that the risk factors of children's habits have a significant association with the incidence of STH infection like not nail trimming (66.7%) and not hand washing (43.8%). This is in accordance with previous study. A study in Ethiopia in 2015 by Shumbej et al. reported that not trimming nails (40.1%) and not washing hands before meals (36%) in children were the main factors significantly associated with STH infection [6].

In this study, the risk factor of hand washing of mother/caregiver is the most influential risk factor for the incidence of STH infection in preschool children compared to the habit of eating uncooked food and nail trimming habit of mother/caregiver. This is in accordance with previous studies in children under five years of age in Ethiopia in 2015 by Aleka et al., where there was a significant association between intestinal parasite infection and the personal hygiene status of the elderly, that is, hand washing and shortening fingernails [2]. However, the results of this study do not match the 2012 Chinese study by Wang et al., where the major factors associated with STH infection are low levels of maternal education and poor preparation of foods such as drinking dirty water or consuming undercooked meat [12].

This study is a study that evaluates risk factors for the incidence of STH infection in preschool children, which is still small study done in Indonesia, especially in North Sumatera where the risk factor that has the most influence is hand washing habit of the mother/caregiver, and it is useful to give input to the local health service that the prevalence of the incidence of STH in children under five years is ≥20% to <50% so that antihelminthic can be administered to preschool children as much as once a year in accordance with Pedoman Pengendalian Kecacingan Kementerian Kesehatan Republik Indonesia 2012 and support WHO deworming program [5, 9]. The limitation of this study is that the data was obtained from questionnaire that may cause recall bias by providing desired answer. Since we were looking for the risk factors causing STH infection in preschool children, we did not do any cognitive test to the study subjects. This could be a suggestion for future research to evaluate the effect of STH infection on short term and long term cognitive function in preschool children.

## 5. Conclusion

Hand washing and nail trimming habit of mother/caregiver and nail trimming habit of children are the risk factors for soil-transmitted helminthiasis in preschool children. Hand washing habit of mother/caregiver is the most influential risk factor for STH infection in preschool children.

## Figures and Tables

**Table 1:** Baseline characteristics.

Characteristics of Subject	
Sex, *n* (%)	
Male	37 (41.1)
Female	53 (58.9)
Age (month), mean (SD)	31.7 (15.50)
Number of family members, mean (SD)	2.2 (1.19)
Number of siblings, mean (SD)	2.4 (1.18)
Weight (kg), mean (SD)	11.6 (2.96)
Height (cm), mean (SD)	83.3 (11.68)
Nutritional status, *n* (%)	
Normoweight	81 (90)
Mild malnutrition	2 (2.2)
Obese	5 (5.6)
Overweight	2 (2.2)
Severe malnutrition	0 (0)
Father's occupation, *n* (%)	
Vegetables farming	78 (86.7)
Rice farming	1 (1.1)
Government employee	0 (0)
Private employee	3 (3.3)
Entrepreneur	8 (8.9)
Others	0 (0)
Father's education, *n* (%)	
Illiterate	1 (1.1)
Elementary school	14 (15.6)
Junior high school	29 (32.2)
Senior high school	41 (45.6)
Diploma/university	5 (5.6)
Mother's occupation, *n* (%)	
Vegetables farming	78 (86.7)
Rice farming	1 (1.1)
Government employee	1 (1.1)
Private employee	2 (2.2)
Entrepreneur	6 (6.7)
Others	2 (2.2)
Mother's education, *n* (%)	
Illiterate	0 (0)
Elementary school	6 (6.7)
Junior high school	20 (22.2)
Senior high school	54 (60)
Diploma/university	10 (11.1)
Total, *n* (%)	90 (100)

**Table 2:** Prevalence, proportion, and intensity of STH infection.

Prevalence, proportion, and intensity of STH infection	*n* (%)
STH infection	
Positive	31 (34.4)
Negative	59 (65.6)
Type of worm	
Ascaris lumbricoides	18 (20)
Trichuris trichiura	4 (4.4)
Hookworm	0 (0)
Mixed infection	9 (10)
None	59 (65.6)
Intensity of *Ascaris lumbricoides*	
Light	22 (24.4)
Moderate	4 (4.4)
Heavy	0 (0)
None	64 (71.1)
Intensity of *Trichuris trichiura*	
Light	13 (14.4)
Moderate	0 (0)
Heavy	0 (0)
None	77 (85.6)
Total	90 (100)

**Table 3:** Risk factors of STH infection in preschool children.

Variables	STH infection		*p* ^*∗*^	PR	95% CI
	Positive	Negative			
	*n* (%)	*n* (%)			
*Habit of children*					
Nail trimming					
Yes	21 (28.0)	54 (72.0)	0.007	5.1	1.57–16.83
No	10 (66.7)	5 (33.3)			
Hand washing					
Yes	3 (11.5)	23 (88.5)	0.003	5.9	1.62–21.89
No	28 (43.8)	36 (56.3)			
Latrine usage habit					
Yes	20 (31.7)	43 (68.3)	0.471	1.5	0.58–3.76
No	11 (40.7)	16 (59.3)			
Barefoot					
No	12 (29.3)	29 (70.7)	0.380	0.7	0.27–1.58
Yes	19 (38.8)	30 (61.2)			
*Habit of mother/caregiver*					
Hand washing					
Yes	4 (13.3)	26 (86.7)	0.004	5.3	1.65–17.12
No	27 (45.0)	33 (55.0)			
Household water source					
Yes	29 (33.3)	58 (66.7)	0.272	4.0	0.35–45.96
No	2 (66.7)	1 (33.3)			
Eating uncooked food					
No	4 (15.4)	22 (84.6)	0.016	4.0	1.24–12.99
Yes	27 (42.2)	37 (57.8)			
Nail trimming					
Yes	19 (27.5)	50 (72.5)	0.018	3.5	1.27–9.66
No	12 (57.1)	9 (42.9)			
Latrine presence					
Yes	30 (35.3)	55 (64.7)	0.656	0.5	0.05–4.22
No	1 (20.0)	4 (80.0)			

^*∗*^Chi-square test.

**Table 4:** The result of multivariate analysis multiple logistic regression for STH infection in preschool children.

Habit	Coefficient	*p*	PR (95% CI)
Step I			
Hand washing of mother/caregiver	1.280	0.079	3.6 (0.86–15.01)
Nail trimming of mother/caregiver	1.276	0.036	3.6 (1.08–11.83)
Eating uncooked food	1.107	0.094	3.0 (0.83–11.05)
Nail trimming of children	1.258	0.070	3.5 (0.61–12.94)
Hand washing of children	1.029	0.188	2.8 (0.90–13.71)
Step II			
Hand washing of mother/caregiver	1.695	0.011	5.5 (1.48–20.03)
Nail trimming of mother/caregiver	1.291	0.032	3.6 (1.11–11.86)
Eating uncooked food	1.083	0.098	2.9 (0.82–10.66)
Nail trimming of children	1.514	0.026	4.6 (1.19–17.31)
Step III			
Hand washing of mother/caregiver	1.762	0.007	5.8 (1.63–20.75)
Nail trimming of mother/caregiver	1.404	0.018	4.1 (1.28–12.97)
Nail trimming of children	1.511	0.022	4.5 (1.24–16.57)
